# The Various Applications of Calcium Phosphate in Medicine

**Published:** 1879-08

**Authors:** 


### Miscellaneous.

### THE VARIOUS APPLICATIONS OF CALCIUM PHOSPHATE IN MEDICINE.

The works of M. Dusart have contributed largely to the spread of the use of phosphate of lime for therapeutic purposes. The importance of this substance is shown by the fact that phosphate of lime is in largest proportion in those animals whose activity is greatest, and whose temperature is highest. Phosphate of lime administered in an insoluble state passes along the alimentary tract, and is for the most part ejected with the fæces without causing any marked changed in the animal economy. An entirely different action takes place, however, when the phosphate is dissolved in lactic acid. Under the form of lacto-phosphate, it stimulates the function of nutrition, whether in the adult or in the infant. In the latter under the influence of this substance the weight of the body undergoes a regular and progressive increase. Whilst exercising this general recruiting influence, lacto-phosphate of lime exerts a special effect upon the osseous system, in which it causes an increase of hardness, or in cases of fracture, consolidation. This double action is the basis for the therapeutic applications of lacto-phosphate of lime. In rickets, M. Dusart finds that in every case in which the diet, though sufficient in quantity, was unsuited to the digestive organs, the addition of lacto-phosphate of lime caused rapid improvement. Very interesting observations upon this subject have been collected in the large hospitals of Paris. In wounds and fractures as in the preceding case lacto-phosphate of lime acts by its invigorating power and by its special action upon osseous tissue, to which it carries the calcareous salt or reparative material. Its employment is chiefly indicated according to Dr. Paquet in those cases in which there exist deeply seated disturbances of the functions of nutrition. The result, which is all but constant, to be attained from this method of treatment is a marked diminution in the usual length of the period of consolidation. Easy pregnancy, constant appetite, a well developed and vigorous child, a rich milk and abundant supply, are the results obtained by M. Dusart from the employment of lacto-phosphate of lime by the mother. Given to the child it keeps up its appetite, favors nutrition, and thus preserves the infant from most of the ailments which are peculiar to the first period of life. In typhoid fever and its convalescent period, in albuminuria, phthisis, diphtheria, etc., the invigorating properties of lacto-phosphate of lime may be used with advantage.—*Gazette Medicale de Paris.*

